# Age Differences in Axial Length, Corneal Curvature, and Corneal Astigmatism in Marfan Syndrome with Ectopia Lentis

**DOI:** 10.1155/2018/1436834

**Published:** 2018-05-02

**Authors:** Jiahui Chen, Qinghe Jing, Yating Tang, Dongjin Qian, Yi Lu, Yongxiang Jiang

**Affiliations:** ^1^Department of Ophthalmology and Vision Science, Eye & ENT Hospital of Fudan University, Shanghai, China; ^2^Key Laboratory of Myopia of State Health Ministry and Key Laboratory of Visual Impairment and Restoration of Shanghai, Shanghai, China

## Abstract

**Purpose:**

To investigate the differences in axial length, corneal curvature, and corneal astigmatism with age in patients with Marfan syndrome (MFS) and ectopia lentis.

**Methods:**

A retrospective case series study was conducted. MFS patients with ectopia lentis were divided into groups according to age. Axial length, corneal curvature, and corneal astigmatism were measured.

**Results:**

This study included 114 MFS patients (215 eyes) with a mean age of 19.0 ± 13.9 years. Axial length differed significantly across age groups in MFS patients (*P* < 0.001), whereas corneal curvature did not (*P* = 0.767). Corneal astigmatism was statistically significant throughout the MFS cohort (*P* = 0.009), but no significant difference was found in young MFS patients (*P* = 0.838). With increasing age, the orientation of the corneal astigmatism changed from with-the-rule astigmatism to against-the-rule or oblique astigmatism (*P* < 0.001). A linear correlation analysis showed weak correlations between age and axial length for both eyes and with corneal astigmatism for the left eye, but there was no correlation between age and corneal curvature.

**Conclusions:**

In MFS, axial length varies with age, corneal curvature remains stable, and corneal astigmatism is higher in young patients and tends to shift toward against-the-rule or oblique astigmatism. Therefore, it is important to consider age when diagnosing MFS with ocular biometric data.

## 1. Introduction

Marfan syndrome (MFS) is an autosomal dominant inherited disease associated with systemic connective tissue disorders, particularly involving the ocular, cardiovascular, and skeletal systems. It is caused by the mutation of fibrillin-1 (FBN1) gene encoding fibrillin-1, which plays important roles in systemic connective tissues and has an integral role in maintaining ocular health [1, 2]. The estimated morbidity rate of MFS was reported to be 4.60–6.81 per 100,000 people [3–5].

Ectopia lentis (EL) affects 30.2%–87.0% of MFS patients [6–14]. Although the incidence of EL varies widely in prior studies, it is the major diagnostic criterion of the 2010 Revised Ghent Nosology [15]. Aside from EL, myopia of >3.0 diopters (D) is a minor ocular criterion for the diagnosis of MFS. Myopia, which is the most prevalent ocular disorder that develops rapidly in early childhood [16, 17], is strongly associated with increased axial length (AL) and decreased corneal curvature, which are common features in MFS patients. A long AL results in myopia; however, a flatter cornea may compensate for increased AL leading to a refractive error of <3.0 D.

Although the phenotype of MFS is becoming well documented in adults, it is incomplete in children, in whom the presence of ectopia lentis and aortic root dilatation are the best and most stable diagnostic features [18]. In young MFS patients, the incomplete phenotype limits the accurate detection of MFS and the timely interventions required to prevent severe complications. Many studies have demonstrated that some ocular biometric characteristics, such as increased AL, reduced corneal curvature, and high corneal astigmatism, have potential diagnostic values in MFS patients [8–10, 19–22]. Increased AL (>23.5 mm), reduced corneal curvature (<41.5 D), and high corneal astigmatism (>1.0 D) are common in MFS, but the differences across age groups have not been determined. Therefore, we investigated the differences in these parameters with age in MFS patients.

Our objective was to evaluate AL, corneal curvature, and the magnitude and orientation of corneal astigmatism in MFS patients with EL in different age subgroups and to determine the clinical diagnostic significance of these parameters for suspected MFS.

## 2. Methods

### 2.1. Ethics

The retrospective case series study was conducted at the Eye & ENT Hospital of Fudan University, Shanghai, China, by searching the medical records of patients treated between March 2012 and November 2017. The study was approved by the Human Research Ethics Committee of the Eye & ENT Hospital of Fudan University, and the research adhered to the tenets of the Declaration of Helsinki. Written informed consent was obtained from all patients.

### 2.2. Subjects

We included patients diagnosed with MFS based on the Ghent-2 criteria [15]. The study group comprised 114 MFS patients (215 eyes) with EL, who came from 15 provinces of China.70.2% of the patients came from the peripheral regions of Shanghai, where our hospital was located. Patients with a history of ocular surgery, corneal disease, uveitis, use of contact lenses within 2 weeks before examinations, or suspected (unconfirmed) MFS were excluded from this study. Both eyes of each subject were included.

### 2.3. Eye Examinations

All patients underwent thorough ophthalmic examinations of both eyes by experienced ophthalmologists. The patients' family and medical history were evaluated before the ocular examinations. We then used slit-lamp examination to determine the position of the lens in different gaze directions under complete pupillary dilation.

AL, corneal curvature, and corneal astigmatism were measured using an IOL Master (Carl Zeiss Meditec, Jena, Germany) or an auto refract keratometer (Nidek ARK-700A). The mean keratometry (Km; in D) was calculated as the mean value of Kmax and Kmin and was taken as the corneal curvature. The corneal astigmatism (AST) was calculated as Kmax–Kmin. The axis orientations of the corneal astigmatism were classified as “with the rule” (WTR) (the steepest corneal meridian was within 90° ± 30°) or “against the rule” (ATR) (the steepest corneal meridian was within 0°–30° or 150°–180°). If the astigmatism was outside these parameters, it was classified as oblique.

### 2.4. Data Analysis

To analyze the differences in these ocular biometric characteristics with age, we divided the MFS patients into five groups by age: G1 (≤10 years old), G2 (11–20 years old), G3 (21–30 years old), G4 (31–40 years old), and G5 (>40 years old). The MFS patients younger than 20 years old were further divided into four subgroups: Y1 (3–5 years old); Y2 (6–10 years old); Y3 (11–15 years old); and Y4 (16–20 years old).

Quantitative data passed the normal distribution test and were presented as the mean ± standard deviation (SD). We used the one-way analysis of variance, Kruskal–Wallis test, and chi-square test, as appropriate, to compare ocular biometric values between different age groups of MFS patients. To determine pairwise differences, a Bonferroni post hoc analysis was conducted when the groups were of equal sizes. Correlations between age and continuous variables were assessed by calculating Pearson's correlation coefficient (*r*). Values of *P* < 0.05 were considered statistically significant. All statistical analyses were performed using SPSS version 22.0 (IBM Corp., Armonk, NY, USA).

## 3. Results

**Figure 1:**
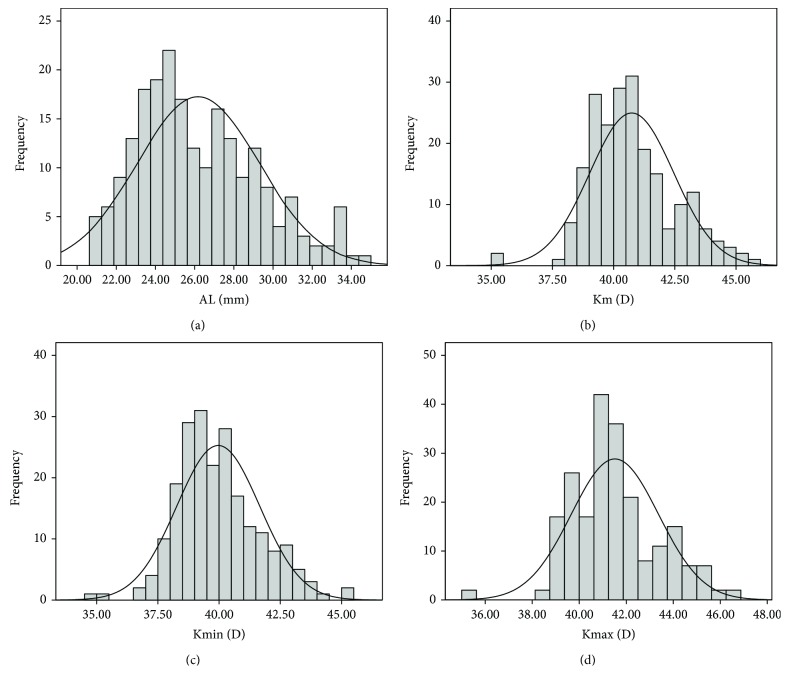
Distributions of axial length (a), Km (b), Kmin (c), and Kmax (d) for Marfan syndrome patients. AL: axial length; Km: mean keratometry; Kmin: minimum keratometry; Kmax: maximum keratometry; D: diopters.

**Figure 2:**
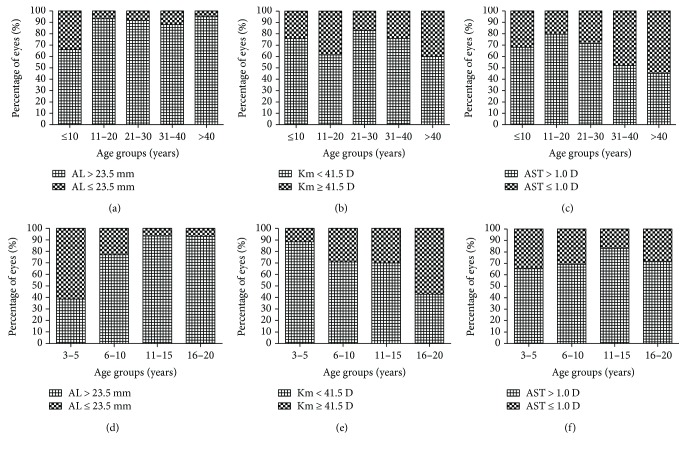
Proportions of increased axial length (a and d), reduced corneal curvature (b and e), and high corneal astigmatism (c and f) in different age groups of Marfan syndrome. AL: axial length; Km: mean keratometry; D: diopters; AST: corneal astigmatism.

The study included 215 eyes of 114 MFS patients with EL. The mean age of the subjects was 19.0 ± 13.9 years (range, 3–54 years), and 50.9% of them were male. The demographic characteristics of the MFS patients are listed in Table 1, and the distributions of elevated parameters are shown in Figure 1. In terms of the minor criteria, we determined the proportions of patients in the different age groups with increased AL, flat cornea, and high corneal astigmatism, which were defined as AL> 23.5 mm, Km < 41.5 D, and AST > 1.0 D, respectively (Figure 2).

### 3.1. Axial Length

**Figure 3:**
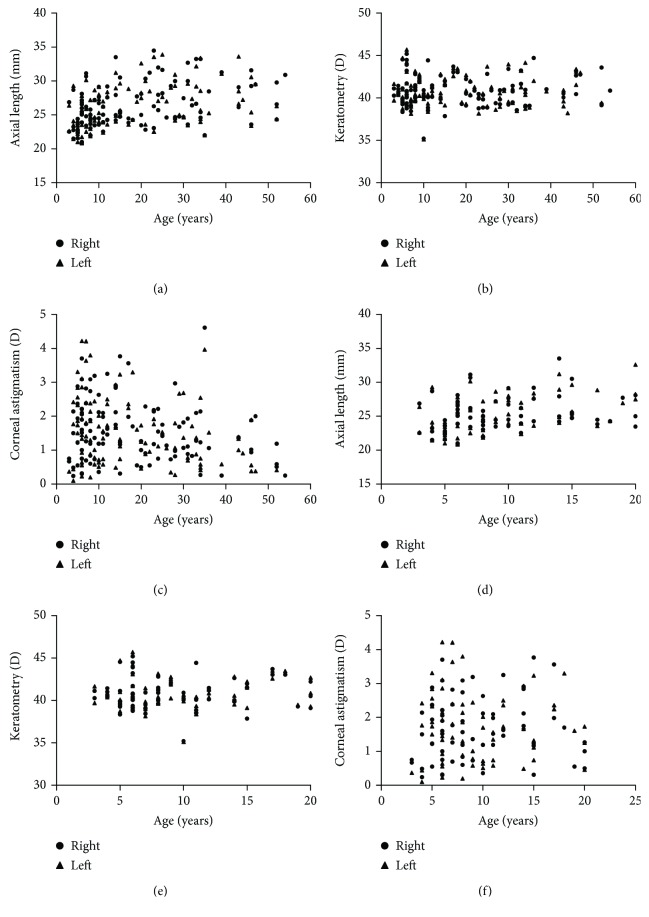
Scatterplots for correlations between the age and AL (a and d), corneal keratometry (b and e), and corneal astigmatism (c and f) in different age groups of Marfan syndrome. D: diopters.

The mean AL of the patients was 26.18 ± 3.11 mm (range, 20.85–34.46 mm) and included 174 eyes (80.9%) with a long AL (>23.5 mm). There were significant differences in AL among the age groups of MFS patients (*P* < 0.001; Table 2). The mean AL was significantly shorter in the G1 group (≤10 years old, 24.79 ± 2.46 mm) than those in the G2, G3, G4, and G5 groups (*P* < 0.05).  Table 3 shows the ocular biometric data for the MFS patients younger than 20 years old. There were significant differences in the mean AL of the Y1 subgroup (3–5 years old) and those of the other subgroups (*P* < 0.05, Table 3). In the young MFS patients, the mean AL and the proportion of AL values > 23.5 mm increased significantly with increasing age (both *P* < 0.001, Table 3, and Figure 2(d)). An abnormally high AL (>23.5 mm) was observed in 60 eyes (65.9%) in the G1 group, in 41 eyes (93.2%) in the G2 group, in 32 eyes (91.4%) in the G3 group, in 22 eyes (88.0%) in the G4 group, and in 19 eyes (95.0%) in the G5 group (*P* < 0.001, Figure 2(a)). A linear correlation analysis showed a weak correlation between age and AL in MFS patients for the right eye (*r* = 0.408, *P* < 0.001; Figure 3(a)) and the left eye (*r* = 0.360, *P* < 0.001; Figure 3(a)).

### 3.2. Corneal Curvature

The mean corneal curvature (Km value) was 40.74 ± 1.72 D (range, 35.13–45.70 D) in the MFS patients with EL. Of the 215 eyes tested, 156 eyes (72.6%) had a flattened cornea, with Km < 41.5 D. The proportions of Km values < 41.5 D in the different age groups are shown in Figure 2(b) (*P* = 0.144). The proportion of Km values < 41.5 D decreased in young MFS patients with age, and the differences were statistically significant (*P* = 0.026; Figure 2(e)). The Km value did not change with age in MFS patients (*P* = 0.767, Table 2; *P* = 0.269, Table 3). No linear correlation was detected between age and corneal curvature (*P* = 0.620 for the right eye, *P* = 0.918 for the left eye, for the total group, Figure 3(b); *P* = 0.686 for the right eye, *P* = 0.620 for the left eye, for the young patients, Figure 3(e)).

### 3.3. Corneal Astigmatism

The mean corneal astigmatism in the MFS patients with EL was 1.55 ± 0.93 D (range, 0.10–4.61 D), and it was higher in the MFS patients < 20 years old (1.70 ± 0.97 D) than in the other patients. Corneal astigmatism differed significantly among the age groups (*P* = 0.009, Table 2), increasing in both the G1 group (age ≤ 10 years) and G2 group (11–20 years) and decreasing with age thereafter in the MFS patients. However, no significant differences were found in the corneal astigmatism of the young MFS patients (*P* = 0.838; Table 3).  Figure 3(c) shows the weak correlation between age and corneal astigmatism in the total MFS patients in the left eye (*r* = −0.274, *P* = 0.004, Figure 3(c)), whereas no linear correlation was detected between age and corneal astigmatism in the young MFS patients (Figure 3(f)).

**Figure 4:**
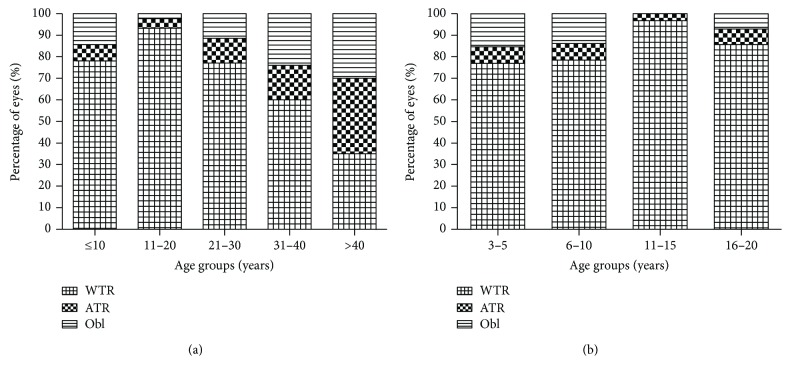
Distributions of the orientations of corneal astigmatism in each age group (a) and subgroup (b) of Marfan syndrome. WTR: with the rule; ATR: against the rule; Obl: oblique.

Among the 215 eyes of MFS patients examined, the type of corneal astigmatism was classified as WTR, ATR, or oblique in 74.9%, 11.2%, or 13.9% of eyes, respectively. The corresponding values were 82.9%, 6.7%, or 10.4% for the eyes of the young MFS patients.  Figure 4 shows the distributions of the orientations of corneal astigmatism in each age group and subgroup. With increasing age, the orientation of corneal astigmatism shifted from WTR astigmatism to ATR or oblique astigmatism (*P* < 0.001, Figure 4(a)), whereas the distribution of corneal astigmatism in the young patient subgroups was primarily WTR astigmatism and did not differ significantly between the subgroups (*P* = 0.395, Figure 4(b)).

## 4. Discussion

In 1981, Maumenee [14] first reported distinctive ocular manifestations of MFS, including globe enlargement, corneal flattening, and lens dislocation. Since then, several studies have demonstrated these ocular biometric characteristics in MFS patients with or without EL [6, 8, 9, 19, 20]. Recently, Kinori et al. [20] reported that the corneas of children with established MFS were flattened to at least the same degree as in adults. It seems that the ocular phenotype of MFS patients displays age-related differences. In this study, we recruited MFS patients with EL to investigate these age differences in AL, corneal curvature, and corneal astigmatism, to provide a better overview of some ocular biometric characteristics of MFS patients of all ages.

In previous studies, AL was reported to be longer in MFS patients with or without EL [6, 20, 21], and we found that 80.9% of MFS eyes with EL had AL > 23.5 mm. The average AL of the patients recruited was 26.18 ± 3.11 mm, which is relatively longer than that of healthy patients in population studies [23, 24] and is consistent with previous reports. Although a longer AL is no longer considered a criterion for the diagnosis of MFS, MFS should be suspected in patients with longer AL. Among the different age groups, the mean AL was significantly longer in older MFS patients, and AL was shorter in the Y1 group (3–5 years old) than in the other MFS patients less than 20 years old. Moreover, a weak correlation between age and AL indicated that AL varies with age in MFS patients with EL. Both the values of AL and the proportions of eyes with AL > 23.5 mm changed with increasing age. Therefore, we propose that AL is less important in the diagnosis of MFS in young patients than in older patients.

Ocular parameters generally have complex and mutual effects on ocular refraction [23, 25]. The length of the globe increases significantly with increasing age, but the anterior segment of the eyeball changes negligibly. The corneal curvature did not differ between the different age groups of MFS patients, whereas the average corneal curvature value (40.74 ± 1.72 D) was low and 156 eyes (72.6%) of our patients had a flat cornea (Km < 41.5 D). Corneal curvature differed significantly in neither the young nor the old MFS patients, which is contrary to a previous study that showed flatter corneas in children with MFS [20]. The Km value did not change with increasing age, and no linear correlation was detected between age and corneal curvature, so corneal curvature remained relatively stable in most MFS patients (mean value < 41.5 D). Therefore, corneal curvature might be useful in the diagnosis of MFS or could represent a warning sign when evaluating patients with suspected MFS.

Several recent studies have highlighted the importance of high corneal astigmatism in the diagnosis of MFS [9, 20]. It has been suggested that any deviation arising from advanced EL and zonule defects, which are very frequent in MFS patients, might increase corneal astigmatism [9, 20, 26]. It has also been proposed that defects in the *FBN1* gene might affect the zonule and corneal connective tissues, resulting in greater corneal astigmatism [9]. When the MFS patients were divided into five groups by age, corneal astigmatism was greater in the young MFS patients and differed significantly between the age groups of MFS patients. Therefore, it is important to take the patient's age into account when assessing corneal astigmatism in MFS patients. Although previous studies have reported that corneal astigmatism is higher in MFS patients with EL than in those without EL [9, 20], MFS patients generally display more corneal astigmatism than healthy people. Therefore, our results imply that higher corneal astigmatism can be considered a positive symptom in the diagnosis of MFS in young patients.

Corneal astigmatism was classified as WTR in most eyes in each subgroup of MFS patients younger than 20 years old. The astigmatic orientation of the cornea in the total cohort of MFS patients tended to shift from WTR to ATR or oblique astigmatism with advancing age, consistent with previous studies of healthy eyes [27, 28]. However, the number of patients in our study was relatively small, which limited our capacity to detect age-related differences in corneal astigmatism. The age-related shift in astigmatism orientation is believed to involve reduced lid tension, increased intraocular pressure, age-related changes in extraocular muscle tension, and changes to the corneal structure [29]. Toric intraocular lenses (IOLs) are perhaps not beneficial for patients with high corneal astigmatism when we consider the shift from WTR to ATR with increasing age, because toric IOLs will not correct corneal astigmatism unless they are correctly aligned along the steep meridian [30]. Therefore, toric IOLs may be unsuitable in MFS patients with high corneal astigmatism, especially young children with EL.

In the revised Ghent criteria, myopia > 3.0 D is a minor ocular criterion for the diagnosis of MFS [15]. However, myopia is the most prevalent ocular disorder in the world, developing rapidly in early childhood and affecting many healthy people. We know that AL is strongly associated with the development of myopia [17]. However, a refractive error of <3.0 D in patients with MFS may be accompanied by corneal flattening. Therefore, myopia > 3.0 D may not be diagnostically useful in children with MFS, despite increased AL. Corneal flattening and increased AL are probably more relevant to the diagnosis of MFS than myopia. Furthermore, advanced EL and zonule defects, which are very frequent in MFS patients, will result in higher myopia and astigmatism [9, 20]. Therefore, a suspicion of MFS should be considered seriously in children with high corneal astigmatism.

There are two limitations to our study. First, our cohort of MFS patients all had EL, which prevented us from detecting differences in patients without EL for early diagnosis. Second, we must acknowledge that not all the patients diagnosed with MFS underwent genetic testing for confirmation. However, it is necessary to assess the ocular characteristics in MFS patients in order to better understand the age differences of this disease.

In conclusion, AL varies with age and MFS should be suspected in older patients with long AL; high corneal astigmatism appears to have utility in the diagnosis of MFS in young patients, and reduced corneal curvature may also be a useful marker in the diagnosis of MFS. With advancing age, the orientation of corneal astigmatism in MFS patients tends to shift from WTR astigmatism to ATR astigmatism or oblique astigmatism. Therefore, age should be considered in the diagnosis of patients with suspected MFS. We propose that AL, corneal curvature, and corneal astigmatism should be measured in the clinical context for patients in whom MFS is suspected based on ocular biometric data.

## Figures and Tables

**Table 1:** Demographic characteristics of the Marfan syndrome patients with ectopia lentis.

Subjects (eyes)	114 (215)
Mean age (range) (years)	19.0 ± 13.9 (3–54)
Gender (female : male)	56 : 58
Right : left	108 : 107
AL (range) (mm)	26.18 ± 3.11 (20.85–34.46)
Kmin (range) (D)	39.96 ± 1.70 (34.87–45.42)
Kmax (range) (D)	41.52 ± 1.86 (35.38–46.75)
Km (range) (D)	40.74 ± 1.72 (35.13–45.70)
AST (range) (D)	1.55 ± 0.93 (0.10–4.61)

AL: axial length; Kmin: minimum keratometry; Kmax: maximum keratometry; Km: mean keratometry; D: diopters; AST: corneal astigmatism.

**Table 2:** Demographic characteristics of the Marfan syndrome patients with ectopia lentis among different age groups.

Age group (years)	G1 (≤10)	G2 (11–20)	G3 (21–30)	G4 (31–40)	G5 (>40)	*P* value^∗^
Eyes (*N*)	91	44	35	25	20	
Male/female	21/26	12/10	10/9	9/5	6/6	0.759
Right/left	45/46	22/22	19/16	12/13	10/10	0.990
AL (mm)	24.79 ± 2.46	26.32 ± 2.63	27.58 ± 3.34	27.75 ± 3.69	27.82 ± 2.85	<0.001
Km (D)	40.73 ± 1.84	40.90 ± 1.65	40.46 ± 1.45	40.69 ± 1.77	41.01 ± 1.75	0.767
AST (D)	1.68 ± 1.00	1.74 ± 0.90	1.44 ± 0.67	1.36 ± 1.08	1.00 ± 0.55	0.009

AL: axial length; Km: mean keratometry; D: diopters; AST: corneal astigmatism; ^∗^*P* values represent general differences among groups.

**Table 3:** Demographic characteristics of the young Marfan syndrome patients with ectopia lentis among different age subgroups.

Age groups (years)	Y1 (3–5)	Y2 (6–10)	Y3 (11–15)	Y4 (16–20)	*P* value^∗^
Eyes (*N*)	26	65	30	14	
Male/female	6/7	15/19	8/7	4/3	0.890
Right/left	13/13	32/33	15/15	7/7	1.000
AL (mm)	23.55 ± 2.11	25.28 ± 2.43	26.31 ± 2.67	26.36 ± 2.66	<0.001
Km (D)	40.59 ± 1.43	40.78 ± 1.99	40.56 ± 1.49	41.63 ± 1.78	0.269
AST (D)	1.55 ± 0.93	1.73 ± 1.03	1.78 ± 0.88	1.68 ± 0.96	0.838

AL: axial length; Km: mean keratometry; D: diopters; AST: corneal astigmatism; ^∗^*P* values represent general differences among subgroups.
